# Effects of environmental enrichment on cognitive performance of pigs in a spatial holeboard discrimination task

**DOI:** 10.1007/s10071-015-0932-7

**Published:** 2015-10-31

**Authors:** Charlotte G. E. Grimberg-Henrici, Paul Vermaak, J. Elizabeth Bolhuis, Rebecca E. Nordquist, F. Josef van der Staay

**Affiliations:** Emotion and Cognition Group, Department of Farm Animal Health, Faculty of Veterinary Medicine, Utrecht University, Utrecht, The Netherlands; Adaptation Physiology Group, Department of Animal Sciences, Wageningen University, Wageningen, The Netherlands; Applied Biology, HAS, University of Applied Sciences, Den Bosch, The Netherlands; Brain Center Rudolf Magnus, UMC Utrecht, Utrecht, The Netherlands; Institute of Animal Breeding and Husbandry, Christian-Albrechts-University, Kiel, Germany

**Keywords:** Reference memory, (General) working memory, Spatial holeboard task, Salivary cortisol, Environmental enrichment, Pig (*Sus scrofa*)

## Abstract

This study investigated the effects of environmental enrichment on the cognitive performance of female conventional farm (growing) pigs in a spatial holeboard task. Ten pairs of littermates matched for weight were used. From each litter, one piglet was randomly assigned to a barren environment; the other was assigned to an enriched environment from 4 weeks of age. The enriched environment was double the size of the barren environment, had a floor covered with straw, a rooting area filled with peat, and one of the four different enrichment toys which were exchanged daily. Starting at 11 weeks of age, all pigs were tested in a spatial holeboard discrimination task in which 4 out of 16 holes were baited. Furthermore, basal salivary cortisol levels of all pigs were determined after the end of all testing. All pigs were able to acquire the pattern of baited holes (acquisition phase, 40 trials) and the diagonally mirrored pattern (reversal phase, 20 trials). During the acquisition phase, the reference memory performance of the enriched-housed pigs was better than that of their barren-housed littermates, i.e. they reduced visits to the unbaited set of holes. During the reversal phase, enriched-housed pigs had a better general working memory performance than the barren-housed pigs as indicated by reduced revisits to holes already visited during a trial, irrespective of whether they were of the baited or the unbaited set. The enriched-housed pigs also searched for the hidden bait faster during both phases. The environments did not affect basal salivary cortisol levels. In conclusion, environmental enrichment slightly improved the cognitive performance of pigs in a spatial learning task. We hypothesise that the long period of habituation to and testing in the holeboard acted as enrichment that partially reduced the effects of barren housing.

## Introduction

Environmental enrichment is believed to satisfy the behavioural needs of pigs to explore and forage and to help the animals to adapt to their environment (Ferguson 2014). Behaviours of pigs housed in barren or enriched environments differ, reflected in different time budgets. Pigs housed in barren environments were less active, less explorative and showed less play behaviour (Beattie et al. 2000b; Bolhuis et al. 2005). Furthermore, they differ with regard to the diversity of their behavioural repertoire (Wemelsfelder et al. 2000), the development of oral manipulative behaviours directed at mates (Bolhuis et al. 2005; van de Weerd et al. 2005), the level of aggression during social interactions (Beattie et al. 2000b), and physiologically concerning their stress response (de Jong et al. 1998, 2000; Beattie et al. 2000a, b; Geverink et al. 2003; van der Staay et al. 2010). Living in a barren environment can be stressful for pigs (Beattie et al. 2000a; de Jong et al. 2000; van der Staay et al. 2010). Pigs housed in barren environments had higher adrenal weights at slaughter (Beattie et al. 2000a), hypothesised to be due to a chronic activation of the hypothalamic–pituitary–adrenal (HPA) axis. Similar results were found in tethered sows, compared with loose sows: tethered sows had heavier adrenal glands at slaughter (van der Staay et al. 2010). Activation of the HPA axis has been found to increase the release of cortisol by the adrenal glands (Selye 1936). However, more recent studies found that chronic stress can also lead to hypocortisolism (Mason et al. 1968; Natelson et al. 1988; de Jong et al. 2000; Geverink et al. 2003). de Jong et al. (2000) confirmed these findings and showed that barren-housed pigs had blunted circadian rhythms in basal cortisol. Enriched-housed pigs had higher baseline cortisol concentrations at 22 weeks of age compared to barren-housed pigs. Similar results were reported by Geverink et al. (2003). Stall-housed gilts had blunted circadian patterns in cortisol concentrations compared to group-housed gilts that were provided with more space, straw, and contact with other gilts.

The hippocampus plays an important role in stress responses. The hippocampus contains high concentrations of glucocorticoid receptors and is responsible for the negative feedback regulation of the HPA axis to restore homoeostasis (Sapolsky 1985). In addition, the hippocampus is involved in spatial navigation and long- and short-term memory (Chiba et al. 1994; Pothuizen et al. 2004). Stress can have negative consequences for hippocampal functioning (Sapolsky 1985). Laughlin et al. (1999) investigated the spatial performance of pigs in a foraging task in an eight-arm radial maze while the pigs were exposed to different stressors during the trials. They found that even mild stress can impair spatial performance of pigs.

Enrichment has stress-reducing effects in pigs (de Jong et al. 1998, 2000; Geverink et al. 2003) that could result in improvement of the cognitive performance. Several rodent studies have shown that enrichment provided by running treadmills, climbing material and toys resulted in better spatial learning performance (Nilsson et al. 1999; Leggio et al. 2005). This effect may be mediated through the hippocampus, as enrichment also produces hippocampal alterations such as stimulation of the neurogenesis in the dentate gyrus (van Praag et al. 1999). In the parietal cortex, enriched animals also had longer dendritic trees, and dendrites with a higher number of nodes and intersections (Leggio et al. 2005).

With regard to commercial pig husbandry, the capability of an animal to adapt to its environment is an important factor for animal welfare (Ohl and van der Staay 2012). A reduced adaptive capacity implies chronic stress for the animal when adaption to the environment is needed (Weiss 1971). Pigs on intensive farms are exposed to several stressful situations during their life, e.g. changes in housing systems, equipment, types of feed, introduction into groups of unfamiliar group members and various human handlers. New techniques have been developed to improve the welfare of intensive housed pigs and thus to give them more possibilities to adapt to their environment. These techniques may well function as enrichment, presumably an improvement in welfare, but it is important to bear in mind that the adaptations required from pigs in intensive farming remain within the adaptive capacities of the animals. Some new techniques rely on the cognitive ability of pigs. For instance, feeding machines have been developed that give an auditory cue indicating meal availability (Ernst et al. 2005), where each pig learns to respond to a distinct acoustic cue. Stables have also been designed that are divided into different areas for rest, activity, feeding, drinking, comfort and defecation (de Greef et al. 2011). Pigs need to have the potential and opportunity to learn to use equipment that makes use of their cognitive capacity, and to use spatial memory to distinguish different areas to use the stable in a desired and effective way.

The aim of the present study was to investigate the cognitive performance of pigs that were housed in a barren or an enriched environment from weaning. The pigs were tested in a spatial holeboard discrimination task for assessing their spatial learning and memory (Arts et al. 2009; Gieling et al. 2012, 2013). This task, with hidden food rewards as appetitive stimuli, stimulates behaviours that resemble natural foraging in pigs (Westlund 2014). Starting at 11 weeks of age, they were trained in the holeboard setup twice a day to a total of 40 acquisition trials, followed by a total of 20 trials on a reversal of the original configuration of baited holes. The enrichment we used was more elaborate than that in a previous study where enrichment only affected working memory (Bolhuis et al. 2013). We therefore hypothesised that pigs from the enriched environment would perform profoundly better in the holeboard task than pigs from the barren environment, in both working memory and reference memory, and that the barren-housed pigs would experience more stress, as indicated by their basal salivary cortisol level at the end of the study.

## Materials and methods

The experiments were approved by the Animal Care and Use committee of Utrecht University and were conducted in accordance with EU directive 86/609/EEC.

### Animals and housing

Duroc × (Terra × Finnish Landrace) pigs from the farm at Utrecht University were used. These pigs were born and kept under conditions commonly found in Dutch pig husbandry. These conditions are broadly described in documents of the Dutch Government (2015) and of the IKBNV (2015). Note that both documents are in Dutch.

Two days before weaning 20 healthy piglets were selected from ten different litters. The two female piglets closest to the average weight in each of the ten litters were selected. Per litter, one of the two piglets was randomly assigned to a barren environment, and the other was assigned to an enriched environment. At 4 weeks of age, the animals were weaned and moved to the experimental housing unit. All ten animals for each condition were housed together. The two environments (barren and enriched) were located side by side in a naturally ventilated stable with natural light from large skylights. The two groups of animals could not see, but could hear and smell one another. The stable temperature was registered daily and ranged between 8 and 20 °C. A radio played popular music from a radio station 12 h per day between 7:00 and 19:00 hours.

The barren environment had a concrete floor and measured 400 × 500 cm. It contained a covered pig nest (130 × 360 cm) with a rubber mattress and two heating lamps, a rubber bite rod, and a drinking and feeding place.

The enriched environment was twice as large, measuring 850 × 530 cm and contained a rooting area of 360 × 270 cm filled with peat. New peat (150 L) was added weekly. A layer of straw bedding covered the concrete floor. Fresh straw was added daily (approximately two heaped wheelbarrows). Furthermore, one of the four different enrichment toys was provided—wooden sticks, balls, jute bags and ropes—which changed randomly daily. The pen contained a covered pig nest with straw bedding (130 × 360 cm) and two heating lamps, two drinking places, a feeding place and two rubber bite rods.

Feed and water were available ad libitum until 2 days before holeboard testing started. Thereafter, to increase motivation to seek the hidden bait in the holeboard task, the pigs were fed 1/3 of the daily feed amount at approximately 7:30 hours in the morning before testing and 2/3 of the daily feed after testing at approximately 16:00 hours.

### Testing area and holeboard apparatus

**Fig. 1:**
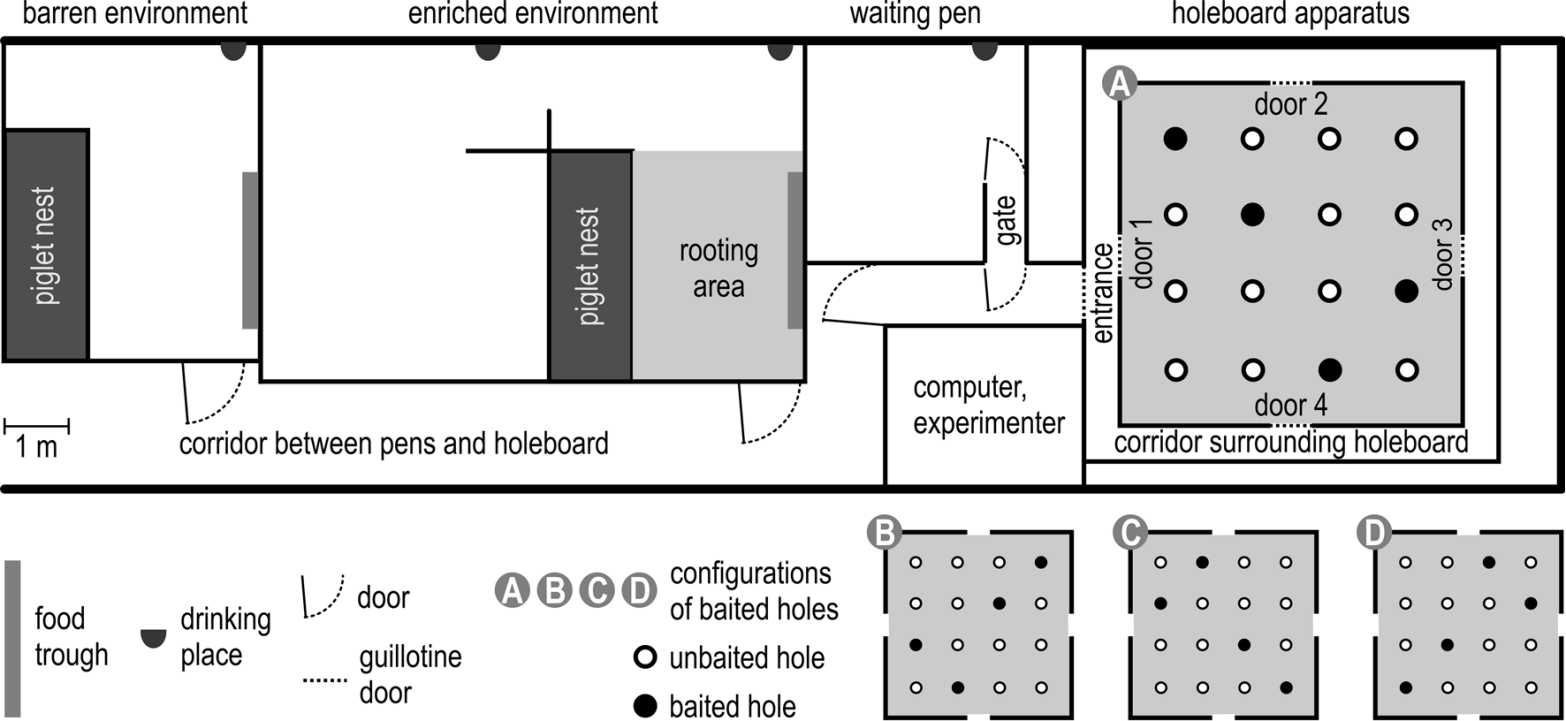
Bird’s-eye view of the spatial cognitive holeboard apparatus for pigs (*A*) and the adjacent housing facilities with enclosures containing barren or enriched environments. Pigs are housed in groups of ten animals in either the barren or enriched environment. The position of the entrance door to the corridor surrounding the holeboard, and the four guillotine doors providing access to the test arena are shown. In *A*, *B*, *C* and *D*, the four different patterns of baited holes are marked by *black dots*. The same configuration has previously been used in rat (van der Staay et al. 1990) and pig studies (Bolhuis et al. 2013)

The barren pen, the enriched pen, a small pen which was used as waiting area before testing and the testing apparatus all were located in the research stable, connected by a corridor (see Fig. 1). During testing, all pigs of one pen were let through the corridor into the waiting pen (343 cm × 273 cm). The last pig tested in a group had a maximum waiting time of 45 min, decreasing to 20 min during the course of the experiment. When the enriched-housed pigs were tested, different enrichment toys—wooden sticks, balls, jute bags and ropes—were provided in the waiting pen. In the waiting pen, a little gate (127 cm × 64 cm) was located in which the next to be tested pig waited. Then the pigs were led individually into the testing apparatus. During testing, in the holeboard apparatus, a pig was able to hear and smell, but not to see, its pen mates and the pigs of the other group.

The testing apparatus was a cognitive holeboard especially constructed for pigs (manufacturer: Ossendrijver BV, Achterveld, the Netherlands). The holeboard arena measured 535 × 535 cm (see Fig. 1A). It had a blue slatted floor and grey synthetic, 80-cm-high walls. The arena was surrounded by a corridor (width 56 cm). Via guillotine doors in the centre of each of the four sidewalls of the arena (width 68 cm), the pigs could enter the holeboard. The whole testing apparatus was elevated 30 cm above the floor.

The test arena was provided with a 4 × 4 matrix of food bowls (space between bowls 95 cm, space between wall and bowls 73 cm). Each food bowl had a false bottom under which three M&M’s^®^ (sugar-coated chocolate candies) were placed to control for the smell of the bait, emanating from the holes, i.e. to prevent pigs from locating the baited bowls based on smelling the rewards. These M&M’s were replaced daily. To prevent the pigs from locating rewarded food bowls by seeing the rewards, each bowl was covered with a red synthetic ball (JollyBall^®^ Dog Toy, diameter 24 cm, weight 400 g). The pigs had to lift the ball with their snout to reach and consume the reward. When the pig retracted its head, the ball fell back into its original position to cover the food bowl again.

### Habituation and behavioural testing

The experiment took place over a period of 12 weeks. After weaning at 4 weeks of age, the pigs were allowed to habituate to their new pen mates and environment. During the first 5 days, the pigs were left undisturbed in their pens. Then, the pigs were gradually habituated per pen as a whole group to their handlers, the testing rooms and the testing apparatus. The handling training took 3 weeks with two daily 30-min sessions. During handling training, the pigs were free to spend time with the handler. In the beginning, the handler fed the pigs chocolate raisins, which are very soft and easy to eat, in order to lure the pigs to approach the handler and to have the chance to stroke the pigs. After 5 days, the handler switched to M&M’s to habituate the pigs to the M&M’s for later testing in the holeboard. Five days later, the handler stopped feeding M&M’s and stroked the pigs as much as possible during habituation sessions. The habituation to the testing room and the testing apparatus lasted 1 week with two daily sessions of 30 min per pen.

Then, a 3-week pre-training phase started, when the pigs were 7 weeks old. The pigs were trained to lift the balls to find the rewards. During the pre-training, all holes were always rewarded (M&M’s). For the first 3 days, the pigs were habituated to stay in the testing apparatus in groups of five pigs twice a day for 30 min. On the first training day, all guillotine doors of the holeboard were open. During the following two training days, only two doors were open (1 and 3; 2 and 4). Then the pigs were habituated in pairs for 5 days twice a day for 15 min. On the first 2 days, two doors were open during training; thereafter, only one of the four doors was open. Finally, the pigs were trained alone for 1 week twice a day until all rewards were consumed or for a maximum of 10 min. Every day another door was opened.

The pigs started with formal testing in the holeboard at the age of 11 weeks. Testing was divided into two phases: an acquisition and a reversal phase. Each pig was assigned to its own configuration of rewarded holes. Every configuration consisted of four rewarded holes out of 16 holes. In each rewarded hole, the pig could find one M&M’s. For the 20 pigs, four different configurations were used as described by Bolhuis et al. (2013) (see Fig. 1A–D). Each pig was tested in the holeboard task once in the morning and once in the afternoon (spaced trials).

The order in which the pigs entered the test arena changed randomly every trial. The entrance door to the holeboard (1–4; see Fig. 1A) was chosen randomly for each trial for each pig. During the acquisition phase, pigs received 40 trials within 4 weeks. In the reversal phase, each pig was tested for 20 trials within 2 weeks. The configuration for the reversal was the diagonally mirrored configuration which was used in the acquisition (Fig. 1; patterns acquisitions—reversal: A–C, B–D, C–A or D–B). The animals were tested on working days.

During testing (acquisition and reversal), the order of visited holes, the number of rewarded and unrewarded visits, the number of revisits of rewarded and unrewarded holes, the number of consumed M&M’s, the latency to the first visit of a hole and the total trial duration were recorded by two observers. The trial started when a pig entered the testing arena with both forelegs. A trial ended when the pig had visited all four rewarded holes or when 5 min had elapsed, whichever event occurred first. A visit was scored when the pig lifted the ball with its snout and an opening between the ball and the bowl was visible.

Faeces and urine were removed, and the apparatus was cleaned with water after a pig was tested.

### Saliva collection and basal cortisol analysis

Saliva was collected once when the pigs were approximately 119 days old. The saliva was collected at the peak of the circadian rhythm of salivary cortisol of pigs at 10:00 hours to account for circadian variability (Ruis et al. 1997). The pigs were allowed to chew on two large cotton swabs (Heinz Herenz, Hamburg, Germany, Cotton Swabs 150 × 4 mm WA 2PL) until the sticks were fully moist (around 30–60 s). Swabs were placed in tubes (Salivette, Sarstedt, Germany) and stored on ice. In the laboratory, at 11:30 hours, the saliva samples were centrifuged for 10 min at 400×*g* and stored at −20 °C until analysis. Salivary cortisol was determined using a radioimmunoassay kit (Coat-a-Count Cortisol TKCO, Diagnostic Products Corporation, Apeldoorn, the Netherlands) adapted for measuring pig salivary cortisol as previously described by Ruis et al. (1997). To avoid inter-assay differences, all samples were assayed on the same day in duplicate.

### Statistical analysis

For the pre-training phase of the holeboard task, the number of different holes visited—the number of holes that were visited at least once during a trial, with a maximum score of 16—was determined per day for the last 6 days. This measure can be taken as an index for how elaborately a pig negotiated the holeboard.

For the acquisition and reversal phase of the holeboard task, three components of spatial memory and three latency/duration measures were calculated (see also van der Staay et al. 2012).

Reference memory (RM) is the number of visits to the baited set of holes divided by the number of visits to all holes. This ratio measure provides an index for the ability of pigs to discriminate between baited and unbaited holes.

Working memory (WM) is the number of rewarded visits divided by the number of visits to the set of holes that is baited at the beginning of the trial. This ratio measure reflects the ability of pigs to avoid revisits to the set of holes baited at the beginning of a trial.

General working memory (GWM) is the number of different holes visited divided by the total number of visits. GWM is a WM measure with respect to all holes. It reflects the ability of pigs to avoid revisits to holes already visited during a trial, irrespective of whether they were of the baited or the unbaited set.

Latency to the first hole visit (LFV) is the time (s) elapsed between entrance of the test arena and the first contact with a hole (lifting the ball and covering the food);

Inter-visit interval (IVI) is the time (s) between first and last hole visits, divided by (number of hole visits −1), reflecting the time per hole visit (lifting the first ball with the snout).

Trial duration (TD) is the time (s) elapsed to find all baits, or, if the pig did not find all baits, the maximum trial duration.

Block means of four successive trials each were calculated per variable. All analyses were performed using the statistical software SAS (version 9.4, SAS Institute, Cary, NC, USA). Residuals were checked for normality, and all variables expressing latencies or durations, IVI, LFV and TD, were log_10_-transformed to meet the normality assumption.

Effects of the environment on the learning curves of the acquisition phase (ten successive trial blocks) and reversal phase (five successive trial blocks), and on the transition between the acquisition phase and reversal phase [last trial block (10) of the acquisition phase versus first block (11) of the reversal phase], as well as on the growth of the pigs were analysed using a mixed model to account for clustering of piglets within litters and repeated measurements within piglets. For holeboard learning, fixed effects were environment (barren vs. enriched), trial blocks and environment × trial blocks. For the growth curves, fixed effects were environment, age in days and environment × age in days.

In all analyses, a random effect for litter was added, and the correlation of repeated measures within piglets was addressed using an autoregressive(1) covariance structure (SAS PROC MIXED).

The effects of environment on salivary cortisol were analysed with the fixed effect environment and the random effect litter (SAS PROC MIXED).

## Results

### Spatial memory

Before the formal training started, the number of different holes visited was scored during six pre-training days, with all holes baited. Averaged over these 6 days, the number of different holes visited was similar in both groups [barren-housed pigs (mean ± SEM) 14.00 ± 0.95 holes; enriched-housed pigs 14.40 ± 1.03 holes; *F*_1,9_ = 0.09, *P* = 0.7688]. In the acquisition phase, the pigs found all four rewards in about 95 % of the 40 trials (barren 92.75 %; enriched 97.25 %). In the reversal phase, the pigs found all four rewards in about 89 % of the 20 trials (barren 89 %; enriched 90 %). Most incomplete trials, i.e. trials in which the pigs did not find all rewards, occurred early during acquisition and reversal, respectively.

#### Reference memory (RM) (see Fig. 2a)

The enriched-housed pigs showed, on average, a better RM performance than the barren-housed pigs during the acquisition (environment *F*_1,171_ = 5.92, *P* = 0.0160) but not during the reversal phase (environment *F*_1,81_ = 1.50, *P* = 0.2239). RM performance of all pigs improved similarly during acquisition (trial blocks *F*_9,171_ = 49.82, *P* < 0.0001; environment by trial blocks interaction *F*_9,171_ = 0.64, *P* = 0.7575) and reversal (trial blocks *F*_4,81_ = 24.64, *P* < 0.0001; environment by trial blocks interaction *F*_4,81_ = 1.19, *P* = 0.3226). The enriched-housed pigs had, on average, a better RM performance during the transition from acquisition to reversal (environment *F*_1,27_ = 5.04, *P* = 0.0331). The decrease in RM performance from block 10–11 was similar in both groups (trial blocks *F*_1,27_ = 129.65, *P* < 0.0001; environment by trial blocks interaction *F*_1,27_ = 0.71, *P* = 0.4055).

**Fig. 2:**
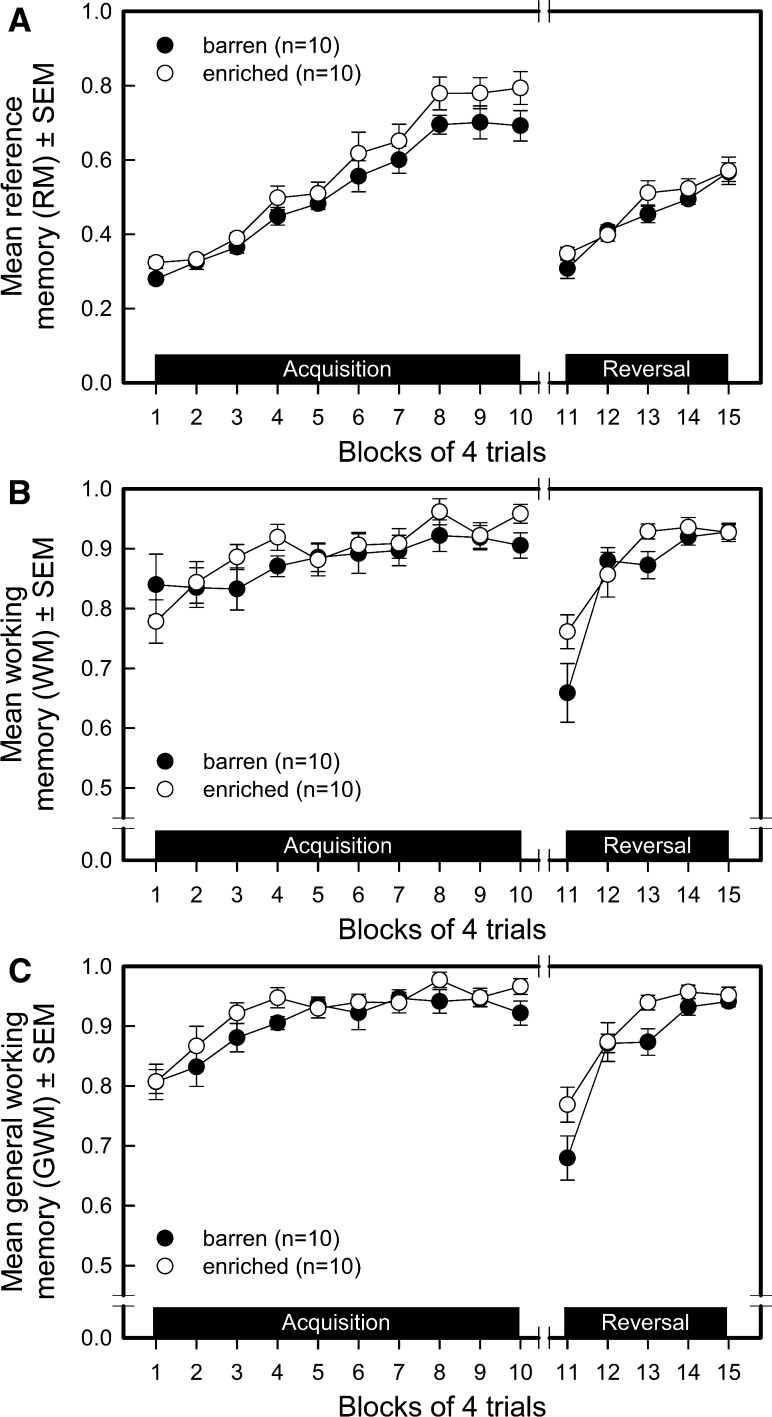
Performance of barren-housed (*n* = 10, *filled circles*) and enriched-housed (*n* = 10, *open circles*) pigs in a spatial holeboard task. The means and standard errors of the means (SEM) for the ten trial blocks of the acquisition and five trial blocks of the reversal phase are depicted for reference memory (**a**), working memory (**b**) and general working memory (**c**)

#### Working memory (WM) (see Fig. 2b)

Environmental enrichment did not affect the average WM performance in the acquisition phase (environment *F*_1,171_ = 1.37, *P* = 0.2429) and tended to improve WM in the reversal phase (environment *F*_1,81_ = 3.45, *P* = 0.0671). The WM performance of both groups improved similarly during acquisition (trial blocks *F*_9,171_ = 3.11, *P* = 0.0017; environment by trial blocks interaction *F*_9,171_ = 0.76, *P* = 0.6504) and reversal (trial blocks *F*_4,81_ = 15.07, *P* < 0.0001; environment by trial blocks interaction *F*_4,81_ = 1.56, *P* = 0.1923). During transition, WM performance was affected by the environment (environment *F*_1,27_ = 4.78, *P* = 0.0377). The pigs from the enriched environment showed a better WM performance than the barren-housed pigs, averaged across trial blocks 10 and 11 (environment *F*_1,27_ = 4.78, *P* = 0.0377). Both groups showed a similar decline in WM performance when presented a new pattern of baited holes (trial blocks *F*_1,27_ = 71.04, *P* < 0.0001; environment by trial blocks interaction *F*_1,27_ = 0.88, *P* = 0.3556).

#### General working memory (GWM) (see Fig. 2c)

During the acquisition phase, the GMW performance tended, on average, to be better in the enriched-housed than the barren-housed pigs (environment *F*_1,171_ = 3.54, *P* = 0.0616). During reversal, the enriched-housed pigs on average performed better on the GWM component of spatial memory than the pigs housed in the barren environment (environment *F*_1,81_ = 7.19, *P* = 0.0089). Both groups of pigs improved their GWM performance similarly during acquisition (trial blocks *F*_9,171_ = 6.81, *P* < 0.0001; environment by trial blocks interaction *F*_9,171_ = 1.06, *P* = 0.3931) and reversal (trial blocks *F*_4,81_ = 22.89, *P* < 0.0001; environment by trial blocks interaction *F*_4,81_ = 1.50, *P* = 0.2113). Enriched-housed pigs showed a better GWM performance than the barren-housed pigs, averaged across trial blocks 10 and 11 (environment *F*_1,81_ = 7.19, *P* = 0.0089). The GWM performance of both groups decreased similarly between the end of the acquisition and the start of the reversal (trial blocks *F*_1,27_ = 80.87, *P* < 0.0001; environment by trial blocks interaction *F*_1,27_ = 0.84, *P* = 0.3678).

### Latencies and durations

#### Latency to first hole visit (LFV) (see Fig. 3a)

The environment had no effect on LFV, averaged over the acquisition sessions (environment *F*_1,171_ = 2.22, *P* = 0.1384) and over the reversal sessions (environment *F*_1,81_ = 0.03, *P* = 0.8675). LFV increased during the acquisition in both groups similarly (trial blocks *F*_9,171_ = 8.62, *P* < 0.0001; environment by trial blocks interaction *F*_9,171_ = 1.30, *P* = 0.2393), whereas it stayed stable during reversal (trial blocks *F*_4,81_ = 1.24, *P* = 0.3008; environment by trial blocks interaction *F*_4,81_ = 1.97, *P* = 0.1065). During transition, the LFV did not change from trial block 10 to trial block 11, nor did enrichment affect this measure (environment *F*_1,27_ = 1.99, *P* = 0.1697; trial blocks *F*_1,27_ = 2.51, *P* = 0.1245; environment by trial blocks interaction *F*_1,27_ = 0.16, *P* = 0.6934).

**Fig. 3:**
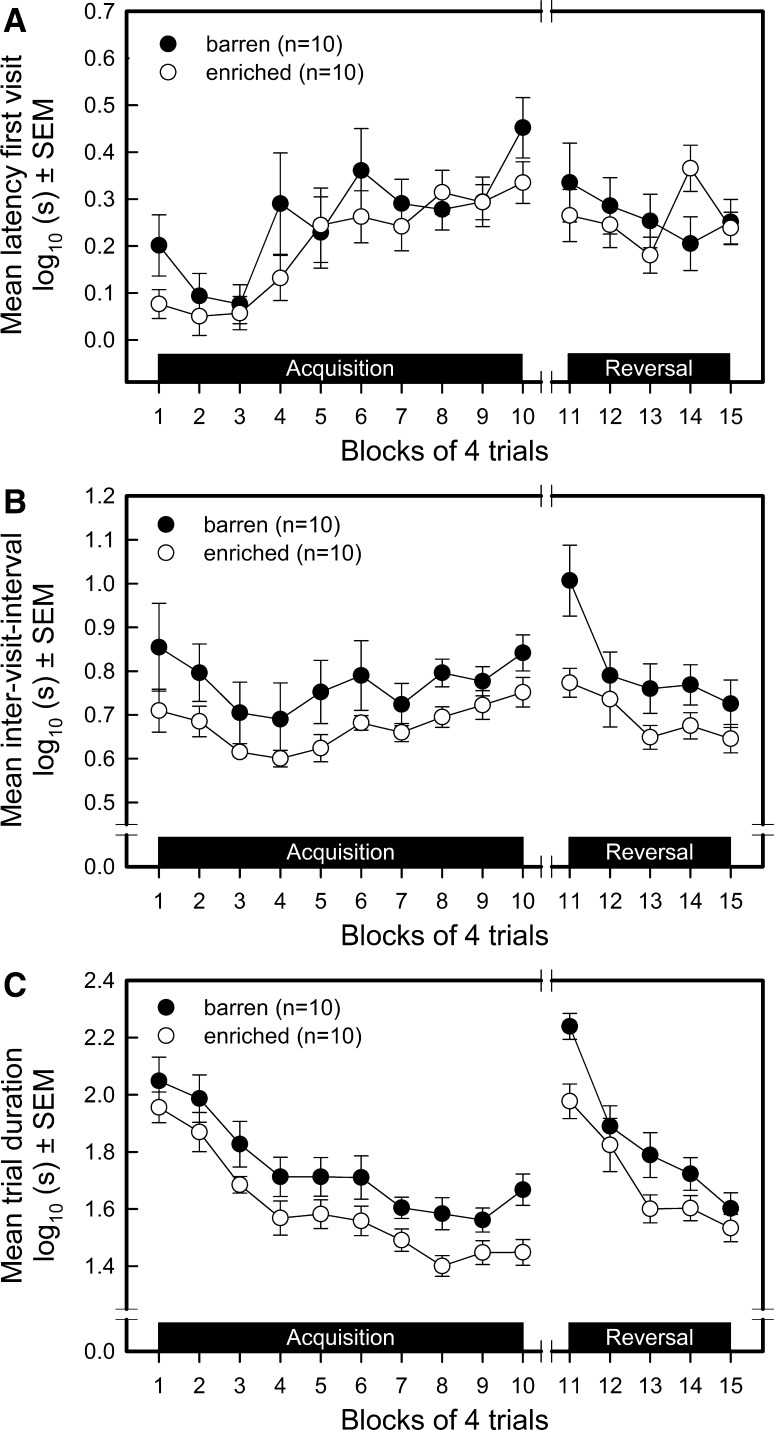
Performance of barren-housed (*n* = 10; *filled circles*) and enriched-housed (*n* = 10; *open circles*) pigs in a spatial holeboard task. Then means and standard errors of the means (SEM) for the ten trial blocks of the acquisition and five trial blocks of the reversal phase are depicted for latency first visit (**a**), inter-visit interval (**b**) and trial duration (**c**)

#### Inter-visit interval (IVI) (see Fig. 3b)

Averaged over trial blocks, the IVI of the barren-housed pigs was longer than that of the enriched-housed pigs during both the acquisition phase (environment *F*_1,171_ = 7.67, *P* = 0.0062) and in the reversal phase (environment *F*_1,81_ = 7.53, *P* = 0.0075). The IVI followed a inversed U shape over trial blocks during acquisition, similarly in both groups of pigs (trial blocks *F*_9,171_ = 3.47, *P* = 0.0006; environment by trial blocks interaction *F*_9,171_ = 0.46, *P* = 0.8990). The IVI decreased over trial blocks during the reversal phase, similarly in both groups (trial blocks *F*_4,81_ = 7.98, *P* < 0.0001; environment by trial blocks interaction *F*_4,81_ = 2.18, *P* = 0.0789). After switching to a new set of baited holes (reversal), the barren-housed pigs initially (in block 11) showed a stronger increase in IVI than did the enriched-housed pigs (environment by trial blocks interaction *F*_1,27_ = 4.38, *P* = 0.0458).

#### Trial duration (TD) (see Fig. 3c)

The average TD during the acquisition phase (environment *F*_1,171_ = 18.27, *P* = 0.0001) and the reversal phase (environment *F*_1,81_ = 9.14, *P* = 0.0033) was longer in the barren-housed than the enriched-housed pigs. Both groups reduced the time needed to find the four baited holes similarly during acquisition (trial blocks *F*_9,171_ = 11.41, *P* < 0.0001; environment by trial blocks interaction *F*_9,171_ = 0.43, *P* = 0.9172) and reversal (trial blocks *F*_4,81_ = 32.56, *P* < 0.0001; environment by trial blocks interaction *F*_4,81_ = 1.59, *P* = 0.1844). The TD increased similarly in both groups after transition, from block 10–11 (trial blocks *F*_1,27_ = 165.06, *P* < 0.0001; environment by trial blocks interaction *F*_1,27_ = 0.25, *P* = 0.6218). During transition enriched-housed pigs needed, on average, less time to find the bait than pigs from the barren environment (environment *F*_1,27_ = 19.97, *P* = 0.0001).

### Physical measurements

#### Growth curves (see Fig. 4a)

The average start weight of the two groups was similar (weight at 28 days for the barren-housed pigs 6.60 ± 0.35 kg, for the enriched-housed pigs 6.93 ± 0.47 kg; *F*_1,9_ = 0.78, *P* = 0.4001) The enriched-housed pigs had a slightly steeper growth curve than their barren-housed littermates (environment *F*_1,135_ = 1.35, *P* = 0.2477; age in days *F*_7,135_ = 270.02, *P* < 0.0001; environment by age in days interaction *F*_7,135_ = 4.46, *P* = 0.0002).

**Fig. 4:**
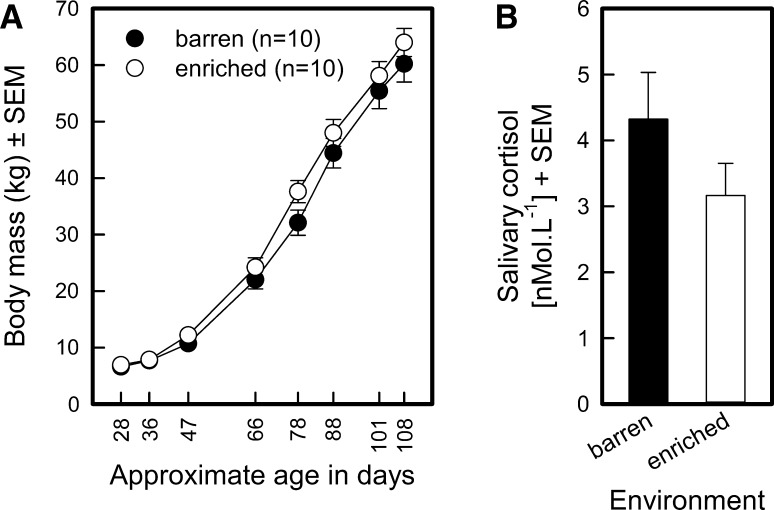
Physical measurements of barren-housed (*n* = 10; *filled circles*) and enriched-housed (*n* = 10; *open circles*) pigs. **a** Growth* curves*. Means and standard errors of the means (SEM) of eight weighing time points are shown. **b** Salivary basal cortisol levels sampled at the end of the study. *Filled bars* represent barren-housed pigs, and *open bars* represent enriched-housed pigs

#### Saliva basal cortisol (see Fig. 4b)

Inspection or Fig. 4b suggests that the mean cortisol level of enriched-housed pigs (3.17 nMol L^−1^) was lower than that of the barren-housed pigs (4.32 nMol L^−1^). This impression, however, was not confirmed statistically (*F*_1,9_ = 1.80, *P* = 0.2124).

## Discussion

Our study provides some support for the hypothesis that enrichment improves cognitive performance in pigs, whereas no evidence was found for an effect of the different environments on basal salivary cortisol as index of stress.

### Spatial memory

During the acquisition and reversal of a spatial holeboard task, enriched- and barren-housed pigs improved their cognitive performance over trial blocks, corroborating earlier studies (Arts et al. 2009; Gieling et al. 2012, 2013; Bolhuis et al. 2013). Both groups of pigs reached their maximum performance approximately in the eighth trial block (after 32 trials) of the acquisition phase for both RM and WM, i.e. faster than the pigs in a study by Gieling et al. (2013) where conventional pigs and minipigs needed about 100 trials before reaching asymptotic, almost error-free levels. During the acquisition phase, enriched-housed pigs showed a better RM performance than their barren-housed littermates. This contrasts with recent findings by Bolhuis et al. (2013) who did not find effects of environmental enrichment on pigs’ RM performance.

Conversely, whereas Bolhuis et al. (2013) found clear improvements of working memory in enriched pigs, in the current study effects of enrichment on working memory measures were marginal, and only statistically confirmed for general working memory during the reversal. In contrast to the current study in which enrichment was applied from weaning at 4 weeks onwards, the pigs in Bolhuis et al. (2013) were enriched from birth, which may have affected the perinatally developing brain differently. Other studies have also reported no effects of environmental enrichment on maze learning (de Jong et al. 2000) or spatial detour learning (Jansen et al. 2009) in pigs.

In these previous studies, enrichment consisted of supplementation of either rooting materials only (Jansen et al. 2009) or rooting materials plus extra space (de Jong et al. 2000; Bolhuis et al. 2013), but the enrichment used in the present study was more elaborate, with more new toys added and a much larger enclosure, which could have affected hippocampal development and functioning. Both increased space allowance and availability of rooting material and objects may have contributed to cognitive performance in different ways. Only few studies in pigs have separated those factors, and for behavioural development and welfare, the provision of rooting materials seems more essential than space allowance (Beattie et al. 1996). It can be speculated, however, that a larger enclosure may also contribute to spatial learning, as has been reported in rodents (Mitsushima et al. 2001).

Barren-housed pigs have been demonstrated to show a changed stress response (de Jong et al. 1998, 2000; Beattie et al. 2000a, b; Geverink et al. 2003; van der Staay et al. 2010), including higher adrenal weights at slaughter due to a chronic activation of the hypothalamic–pituitary–adrenal (HPA) axis (Beattie et al. 2000a; van der Staay et al. 2010). More recent studies found that chronic stress can also lead to hypocortisolism (Mason et al. 1968; Natelson et al. 1988; de Jong et al. 2000; Geverink et al. 2003). The hippocampus plays an important role with regard to stress, and stress can have negative consequences for hippocampal functioning (Radley et al. 2015). The hippocampus is involved in spatial navigation and long- and short-term memory (Chiba et al. 1994; Pothuizen et al. 2004), and is involved in both working and reference memory (Yoon et al. 2008; Conrad 2010). One could hypothesise that barren housing has a negative impact on hippocampal development and thus on spatial memory.

#### Latencies and durations

Neither group reduced their LFV during acquisition nor during the reversal. LFV was in general short, indicating that pigs were motivated to do the task; the LFV showed a small (though significant) increase over time from approximately 2 s in the first trial to 3 s in the last trial. Given these extremely short latency times, this is likely to reflect animals learning during training to walk to a rewarded hole as first hole to visit, rather than visiting the first hole they encountered. Enriched- and barren-housed pigs learned to visit the holes (i.e. IVI) faster and needed less time to complete a trial (i.e. TD) during the course of both the acquisition phase and reversal phase. The enriched-housed pigs were faster to visit holes and completed trials faster than their barren-housed conspecifics. Similar results were found in earlier studies of pigs tested in the holeboard (Gieling et al. 2012, 2013; Bolhuis et al. 2013). This finding is in line with results by Sneddon et al. (2000), who observed that enriched-housed pigs needed less time to find a rewarded container in a T-maze compared to barren-housed pigs.

The improvements and differences of both groups in the present study concerning the IVI may reflect motivational, apart from cognitive, differences between groups. Keuker et al. (2004) suggested that mobility in a holeboard, i.e. the time that an animal spends per hole (in the present study reflected by the IVI), is an index for the level of motivation. Hsia (2004) tested food deprived pigs in a running experiment pigs with low feeding motivation ran slower. Hanmer et al. (2010) investigated enrichment preferences of rats with a runway paradigm. Rats ran faster when they were highly motivated to reach the enrichment object. It could be concluded from the present findings that the enriched-housed pigs showed shorter IVI on average and that they were more motivated to perform the task than pigs from the barren environment. Alternatively, barren-housed pigs may have been less motivated to return to their resident pen. Several studies have shown an increased exploration, i.e. sniffing and rooting, of novel environments (Mendl et al. 1997; de Jong et al. 1998), including maze tasks (Jansen et al. 2009), which has been attributed to the thwarted motivation to explore in their home environment (Wemelsfelder et al. 2000).

### Growth curves

Beattie et al. (2000b) reported that environmentally enriched-housed pigs had higher growth rates and heavier carcass weights at slaughter. In our study, the enriched-housed pigs grew faster than the barren-housed pigs, although the effects on growth were small. All pigs of the present study were housed in a naturally ventilated stable. The temperature in the stable was almost equal to the ambient temperature (ranging from 8 to 20 °C). Both groups had covered nests with heat lamps. The temperature in the nests did not differ between the two environments.

However, the nest and the entire floor in the enriched environment were covered with straw, whereas the barren-housed pigs had only a rubber mattress in the nest and no covering on the concrete floor. Provision of straw bedding may constitute one of the most effective measures for improving pig welfare (van de Weerd et al. 2006; Ferguson 2014). Hayne et al. (2000) observed that pigs housed on a thick layer of straw lay with a more lateral posture, while pigs with low amounts of straw huddled with a sternal posture and piled more than pigs kept on a thick layer of straw. Straw protects pigs against heat loss. Vanheukelom et al. (2011) reported that pigs with access to peat as environmental enrichment grew faster than pigs that did not have access to peat. Both straw and peat stimulate exploratory behaviour and may reduce aggression towards the penmates (Vanheukelom et al. 2011), such as tail biting (van de Weerd et al. 2006).

### Stress and basal saliva cortisol

Pigs from barren and enriched housing did not differ in basal salivary cortisol levels, measured at the end of the study. Belz et al. (2003) found that enriched-housed rats had a lower basal level of adrenocorticotropic hormones and corticosterone compared to rats that were housed without enrichment. A study conducted by van der Staay et al. (2010) found that 4.5-year-old loose housed sows had lower cortisol levels than age-matched tethered sows. Higher cortisosterone levels are associated in rats with impaired cognitive performance (Bodnoff et al. 1995), more metabolic vulnerability of hippocampal cells (Sapolsky et al. 1985), atrophy of CA3 dendrites (Watanabe et al. 1992) and decreased levels of brain-derived neurotrophic factor in the hippocampus (Chao and McEwen 1994). However, these findings are in contrast with more recent studies that found that barren-housed pigs showed lower baseline cortisol level compared to pigs from enriched environments (de Jong et al. 1998, 2000; Geverink et al. 2003), and a blunted circadian rhythm (de Jong et al. 2000; Munsterhjelm et al. 2010). Barren housing conditions can lead to chronic stress which can result in hypocortisolism in animals (Natelson et al. 1988).

In rodent studies, clear differences in cognitive performances and neuronal structures between barren- and enriched-housed animals were found. Enriched-housed rats showed an increased hippocampal neurogenesis (Nilsson et al. 1999; Fares et al. 2013), longer dendrites with more nodes and intersections (Leggio et al. 2005), significantly better spatial performances (Nilsson et al. 1999; Bruel-Jungerman et al. 2005; Fares et al. 2013) and an improved long-term recognition memory compared to barren-housed conspecifics (Bruel-Jungerman et al. 2005).

### Testing as enrichment

Enrichment, i.e. increasing the complexity of the surroundings (Carlstead and Shepherdson 2000), is expected to improve the environment of captive animals and to increase their physiological and psychological well-being (Claxton 2011; Melfi 2013), their biological functioning or their general welfare (Westlund 2014). The “key to successful enrichment is in its complexity and variability (…). These features keep enrichment interesting and novel and encourage animals to interact with their environment” (Laule 2003, p. 970). Enrichment can increase the diversity of behaviours, especially natural behaviour, and decrease abnormal behaviour, as well as improve how the environment is used, i.e. it may increase the ability to cope with environmental challenges (Westlund 2014). The hidden bait as appetitive stimulus encourages natural foraging behaviour in the pigs (Westlund 2014), and stimulates some natural behaviours such as inspecting the holes by lifting the balls and covering the holes with the snout (rooting).

Complex learning and memory tasks may be less suited to assess the effects of environmental enrichment in animals. The pigs in the current study had access to physical activity and enrichment due to the testing in the holeboard. The long-lasting, extended handling and habituation and the subsequent training in the holeboard apparatus may reduce (some of) the negative effects associated with living in a barren environment. Testing interrupted the daily routine, especially for the barren-housed pigs. Tang (2001) showed that rats exposed to a novel environment for 3 min daily after weaning showed better hippocampus-dependent learning. The learning enhancement persisted during ageing. Furthermore, it was found in rats that physical activity results in better spatial learning performance (Fordyce and Farrar 1991; Anderson et al. 2000) and increased hippocampal neurogenesis (van Praag et al. 1999).

Searching for food is one of the pigs’ main behaviours. Under semi-natural conditions, domesticated pigs spend approximately 52 % of the day foraging during the daylight period (Stolba and Wood-Gush 1989). Several studies have shown that pigs are motivated to work for food (Puppe et al. 2007; Arts et al. 2009; Gieling et al. 2012, 2013). Our holeboard apparatus used the natural rooting movements of pigs, which is in itself rewarding for pigs (Studnitz and Jensen 2002; Studnitz et al. 2003). A study by Puppe et al. (2007) showed that pigs trained in a food rewarded learning system, a combination of operant and classical learning, were less active, excited and fearful in an open-field test compared to control pigs. Furthermore, less belly nosing behaviour, a problematic and damaging behaviour seen in pigs in which the nose is pressed and/or rubbed against a conspecific’s belly repeatedly to the point of causing skin damage, was observed to occur less in the trained group than in the control group.

We used positively reinforced behaviour which offers the pig control over its environment, makes the environment predictable, teaches the pig how to use its environment optimally and may increase coping abilities (Young 2003). Moreover, the test procedure may be even more rewarding for the barren-housed pigs, as barren environments have been shown to result in rebound activities in test arenas as a result of thwarted motivation for exploration in the home environment (e.g. Wemelsfelder et al. 2000). Recent studies, moreover, suggest a general higher reward sensitivity in barren-housed animals (e.g. Beckmann and Bardo 2012; Mitchell et al. 2012). It is possible that the effect size of barren housing versus enriched housing is reduced in our test due to an unequal effect of positive reinforcement on the two groups.

## Conclusion

The present study provides some evidence that pigs reared in an enriched environment after weaning show a better cognitive performance in a spatial holeboard task compared to pigs reared in a barren environment. Both groups improved their RM, WM and GWM performance during the acquisition and the reversal phase. Enriched-housed pigs showed a better reference memory performance during the acquisition phase, and a marginally better general working memory performance during the reversal phase. In addition, enriched-housed pigs were faster in the time needed per hole visit (IVI) during acquisition and reversal and needed less time to complete a trial than their barren-housed littermates. The latter finding may be due to a combination of a better spatial memory performance (i.e. fewer errors and consequently less visits per trial) and a shorter time per hole visit. The shorter IVI suggests higher motivation of enriched-housed pigs compared to barren-housed pigs.

The holeboard task is a valid measurement instrument for spatial discrimination learning in pigs. However, it is unclear to what extent the holeboard testing procedure itself could have provided enrichment that could (partially) have counteracted the effects of living in a barren environment and may lead to underestimation of the effects of a barren environment. Therefore, it may be difficult to test effects of different environments on cognitive performance in pigs using longer-lasting, appetitively motivated complex testing procedures.
